# RETRACTION: Effect of *Glycyrrhiza* on the Diuretic Function of *Euphorbia kansui*: An Ascites Mouse Model

**DOI:** 10.1155/ecam/9835052

**Published:** 2026-02-11

**Authors:** Evidence-Based Complementary and Alternative Medicine

RETRACTION: Y. Lin, Y. Zhang, E. Shang, et al., “Effect of Glycyrrhiza on the Diuretic Function of Euphorbia kansui: An Ascites Mouse Model,” *Evidence-Based Complementary and Alternative Medicine*, 2016, https://doi.org/10.1155/2016/7620817.

The above article, published online on 9 May 2016 in Wiley Online Library (https://wileyonlinelibrary.com), and the correction published on 16 December 2018 (https://doi.org/10.1155/2018/5171687) have been retracted by agreement between the Editorial Board and John Wiley & Sons Ltd. The retraction has been agreed following the investigation of figure concerns initially raised on PubPeer [1], whereby similar images were identified with another article [2]. Specifically,

• In Figure 6(b), the G/C‐a panel appears identical to Figure 4(b) GS panel in [2].
• In Figure 6(b), the GS‐1 panel appears identical to Figure 4(b) Model panel in [2].
• In Figure 8(c), the western blots for Mod, G/C‐a, G/C‐s, and GS‐1 appear identical to the western blots for AVPR2 and ß‐actin in Figure 7(c) [2].
• In Figure 7(b), the GS‐1 panel appears to be a magnified and rotated version of Figure 5(c) Model panel in [2].
• In Figure 7(b), the Con panel appears to be a magnified and rotated version of Figure 5(b) GS panel in [2].
• In Figure 7(b), the G/C‐s panel appears to be a magnified, rotated, and flipped version of Figure 5(b) GS/GC_synergy panel in [2].

The authors were contacted and provided unedited data, however, the response did not satisfy our concerns and it is therefore retracted.

The authors were notified about the retraction but did not respond.
